# Tropical rain forest conservation and the twin challenges of diversity and rarity

**DOI:** 10.1002/ece3.705

**Published:** 2013-08-06

**Authors:** Stephen P Hubbell

**Affiliations:** 1 Department of Ecology and Evolutionary Biology, University of California, Los AngelesLos Angeles, California, 90095; 2 Center for Tropical Forest Science, Smithsonian Tropical Research Institute9100 Panama City Pl, Washington DC, 20521-9100

**Keywords:** tropical forest conservation, tropical tree rarity

## Abstract

Data from a global network of large, permanent plots in lowland tropical forests demonstrate (1) that the phenomenon of tropical tree rarity is real and (2) that almost all the species diversity in such forests is due to rare species. Theoretical and empirically based reasoning suggests that many of these rare species are not as geographically widespread as previously thought. These findings suggest that successful strategies for conserving global tree diversity in lowland tropical forests must pay much more attention to the biogeography of rarity, as well as to the impact of climate change on the distribution and abundance of rare species. Because the biogeography of many tropical tree species is poorly known, a high priority should be given to documenting the distribution and abundance of rare tropical tree species, particularly in Amazonia, the largest remaining tropical forested region in the world.

## Introduction: Global Patterns of Tropical Tree Commonness and Rarity

The phenomenon of rarity in tropical tree communities has been known qualitatively in Western scientific circles at least since the writings of Alfred Russel Wallace (1878). However, not until recently have biogeographers and ecologists systematically quantified diversity and rarity in tropical tree communities. Over the last several decades, large-plot tropical forest inventories have been completed that provide quantitative data on tropical tree rarity. This study summarizes the patterns of rarity of tree species in a pan-tropical network of large permanent forest plots, and explores some of the challenges posed by tree rarity and distribution for conservation of tropical tree biodiversity. It relies primarily on the data assembled by the scientists participating in the coordinated global research program on tropical forest dynamics of the Center for Tropical Forest Science (CTFS), on the web at http://www.ctfs.si.edu. However, we also refer to recent findings from an extensive geographical sample of Amazonian tree diversity (ter Steege et al. 2013). The main conclusions from this overview are as follows: (1) tree species diversity in lowland tropical forests is largely due to the presence of rare and very rare species; (2) many of these rare species probably have restricted geographical ranges, contrary to the contemporary view expressed in the “all-species-are-everywhere” hypothesis; and (3) the first two conclusions provide significant challenges for conservation of tropical forest tree diversity, but major uncertainties remain in characterizing the geographic distribution and diversity of rare tropical tree species. How these uncertainties are resolved and the answers to questions about tropical tree rarity have profound practical implications for tropical forest conservation, and should be a high priority for near-term research.

Before proceeding further, it is perhaps useful to be more precise about the usage of the terms *commonness* and *rarity* in this study. Most of the CTFS data discussed here come from inventories of trees in plots of tropical forest that range from 10 to 50 ha in size. In these plots, a convenient comparative rule of thumb is to contrast species that have average abundances of <1 individual per ha with those that have ≥1 tree per ha, and to call the species in the first abundance class rare and those in the second class common. This simple dichotomy is a local-community definition of commonness and rarity, and it does not necessarily say anything about the global abundance of these species – a point to which I will return. Another measure of rarity I use in this study is to contrast the collective abundance of the rarest 50% of the tree species with the abundance of common species that comprise 50% of all tree individuals, in tropical forest plots.

The size and geographic extent of CTFS coverage of tropical forests, although not as extensive as one might ideally like, is nevertheless large, and the CTFS data set provides considerable power to address fundamental questions about tropical tree diversity and rarity on a worldwide basis. The CTFS network now has about 25 plots in tropical Asia, Africa, and Latin America, and the network is expanding into temperate and boreal forests, with a new name, the Smithsonian Institution Global Earth Observatory (SIGEO). The existing tropical plots, many of which are half a square kilometer in size, typically contain between 150,000 and 300,000 trees, saplings, and shrubs. All free-standing plants in the plots >1 cm in stem diameter at breast height (DBH) are individually tagged, mapped and identified, and monitored over time to record recruitment, growth, and mortality. Collectively, the CTFS tropical plots contain over 4,500,000 tagged trees, saplings, and shrubs of about 8500 species. Although the total number of tree species in tropical forests remains unknown, a reasonable estimate from taxonomists is that there are between 25,000 and 50,000 tropical tree species in the world (H. ter Steege, pers. comm.). If this range of estimates brackets the true number, then the CTFS plots contain between a sixth and a third of all tropical tree species.

**Figure 1:**
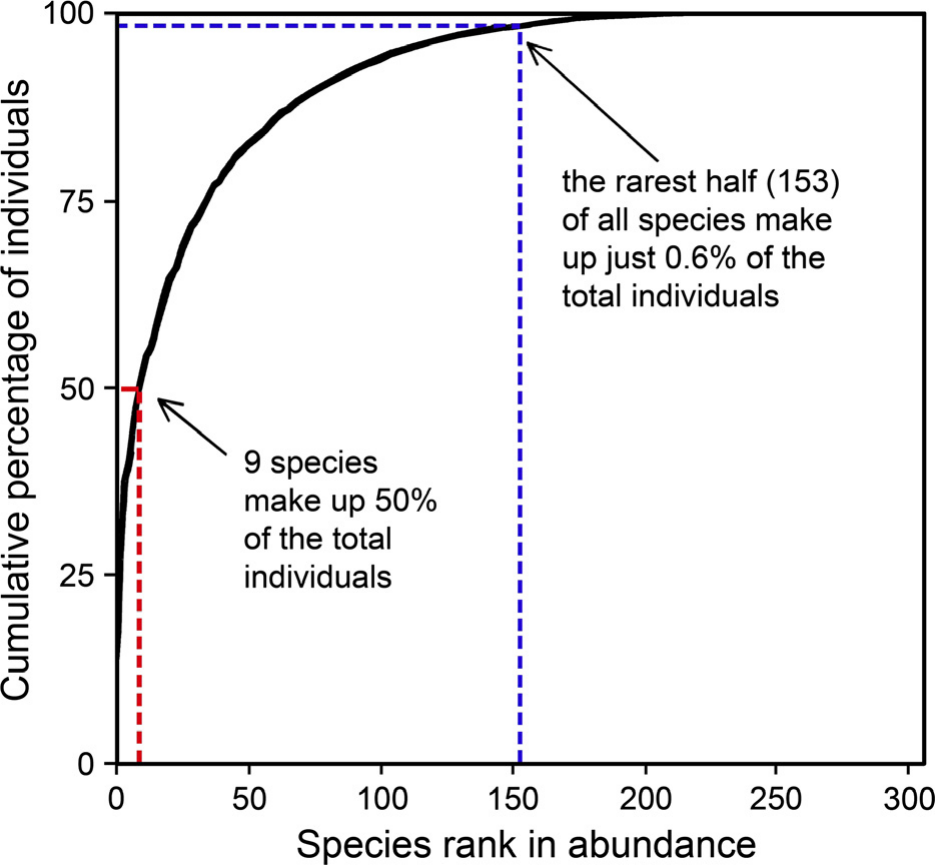
Commonness and rarity of tree and shrub species in the 1982 census of the 50 ha forest plot on Barro Colorado Island, Panama. Cumulative number of individuals (*y* axis) over species ranked in abundance from common (left end, *x* axis) to rare (right end, *x* axis). Of the 306 species, the nine most abundant species make up half of the individuals (about 121,000 individuals). However, the rarest half (153) of the Barro Colorado Island species collectively constitute only six tenths of one percent of all individuals.

What are the general patterns of commonness and rarity across the CTFS tropical plots? Tree species richness in the CTFS plots varies, particularly across differences in annual rainfall. In lowland tropical forests, total species richness varies about six- to eightfold, from a low of about 150–200 species per half square kilometer to a high of over 1300 species. However, on a percentage basis, common and rare species make up similar fractions of species in each of these forests. In one sense, even the most species-rich tropical forests are rather species poor. On average, the top 4.2% ± 2.1% (mean ± 1 standard deviation across plots) of the most abundant species make up half of all the individuals in the CTFS plots. Over all the CTFS plots in tropical forests, just 360 of the 8500 tree species constitute 2.3 million of the 4.5 million individuals. So where does all the tree diversity come from? It comes from the rare and really rare tree species. The rarest *half* (4250 species) of all CTFS species collectively make up just 2.1% ± 2.0% of all individuals, only 95,000 of the total of 4,500,000 trees. For example, consider tree species rarity in the first-established CTFS plot, founded on Barro Colorado Island (BCI), Panama, in 1980 (Hubbell and Foster 1983) (Fig. 1). In the first census, completed in 1982, we encountered 306 species in the 50 ha BCI plot among a total of about 242,000 individuals. Figure 1 shows the accumulation curve of percentage of individuals (*y* axis) summed over species ranked in abundance (*x* axis), from commonest to rarest, left to right. Focusing on the most abundant species, one observes that the nine commonest species make up half of the individuals in the BCI plot. However, consider the rarest half of all BCI species: the 153 rarest species collectively make up only six tenths of one percent of all the individuals.

The mathematics of rarity in the CTFS plots is simply this: 95,000 individuals divided by 4250 species works out to an average of just 22 individuals per species of the rarest half of all species in the CTFS network. Even more dramatic are the numbers of singleton species that have only a single individual in the CTFS network. The average percentage of singleton species per plot is 7.2% ± 3.2%, approximately one in every 14 tree species. Thus, we have the astonishing result that >600 tree species are so rare that they occur only once as a single individual in the entire global CTFS network!

## The Distribution of Rare Tropical Tree Species

From a conservation perspective, one key issue is how the absolute abundance and the geographic range of species are connected. If individual rare species are geographically widespread, their conservation will be an easier task than if many are endemic to relatively small geographic areas. In tropical montane areas, the pioneering work of Alwyn Gentry and others has demonstrated that local endemism is commonplace (Gentry 1982); but in the lowland tropics, the idea has developed that forests are composed mainly of geographically widespread species, even those that are quite rare locally. I dub this perspective the “all-species-are-everywhere” hypothesis. Although not all tropical forest biogeographers and ecologists ascribe to this view, it is certainly the prevailing view; and there is some evidence to support it. For example, Condit et al. (2002) examined beta diversity on local to geographic scales in central Panama and western Amazonia. They characterized beta diversity by the decay in the probability that two randomly chosen trees in forests separated by distance *d* would be of the same species, given a certain dispersal rate by diffusion. They found that this probability declines very quickly over short distances, but then the rate of decay in this probability slows considerably over longer distances, measured up to 1000 km or more. This method for measuring beta diversity, however, is not independent of the abundances of tree species, and is largely driven by widespread, common species. Thus, if widespread species also tend to be more abundant in local forest stands, this method will underestimate the turnover of rare species.

**Figure 2:**
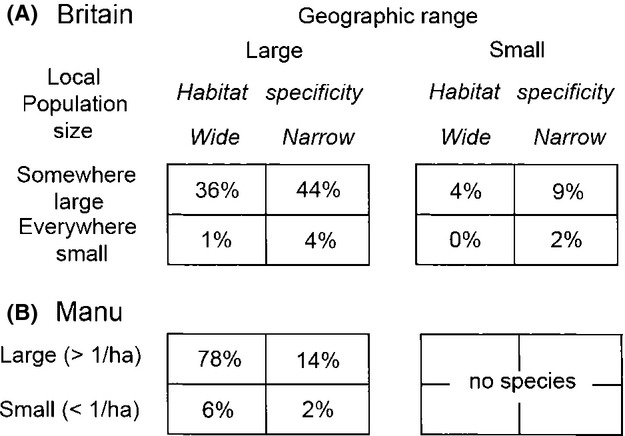
Commonness and rarity patterns reported by Rabinowitz et al. (1986) for species in the British flora (Panel A) and by Pitman et al. (1999) for Manu National Park in Amazonian Peru (Panel B), compared using the qualitative classification scheme of eight forms of rarity proposed by Rabinowitz et al. Rabinowitz et al. reported that 15% of surveyed British plant species had small geographic ranges, but Pitman et al. reported no Manu species in this category. Note that there is a difference in the two methods because Pitman et al. were not able to assess whether local population sizes were “somewhere large” versus “everywhere small” as did Rabinowitz et al., who had complete and relatively detailed range maps of British plant species.

An example of the “all-species-are-everywhere” hypothesis is the study by Pitman et al. (1999), who analyzed tropical tree distributions in 21 forest plots totaling 36 ha along a 20 km section of the Manu River in Manu National Park, in Amazonian Peru. Pitman et al. used the qualitative method for classifying the geographic distribution of common and rare species devised by Deborah Rabinowtz (Rabinowitz et al. 1986). Rabinowitz instructed a group of untutored undergraduates to classify the patterns of distribution of plants in the British flora into eight categories in a 2 × 2 × 2 matrix: Geographic range (large or small); habitat specificity (wide or narrow), and local population size (somewhere large or everywhere small), and she then took a majority vote of the students to classify each species. Rabinowitz et al. reported that the students decided that 85% of species had large geographic ranges (Fig. 2, Panel A). Only 7% of species were rare if one defines a rare species as having everywhere small population sizes. Of this 7%, only 2% of all species had small geographic ranges and everywhere small populations. When Pitman et al. applied the same classification scheme to tree species in the Manu River plots, they obtained a strikingly different result (Fig. 2, Panel B): there were apparently no species that had small geographic range; in fact, nearly three quarters (73%) of all species in the Manu plots were habitat generalists with wide geographic ranges, and two thirds had “large” local populations (>1 individual per ha). The evidence for most species having wide geographic ranges was primarily based on species having large local populations (85% of all species). N. C. A. Pitman (pers. comm.) recently commented by way of clarification that their study was primarily considering species in western Amazonia. However, the question remains whether they really had adequate data to address the distribution of rare and very rare species on large spatial scales across western Amazonia.

**Figure 3:**
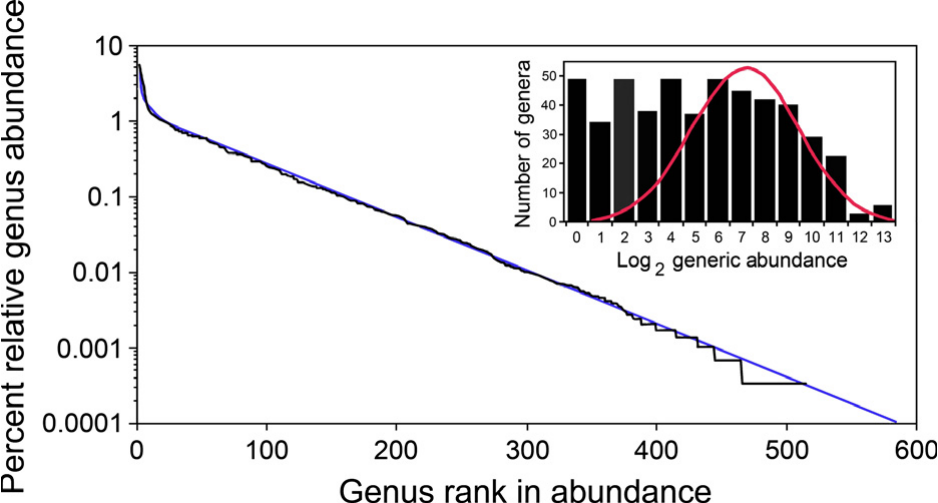
The fit of Fisher's log series to the rank-abundance curve for Amazonian tree genera. After Hubbell et al. (2008), based on data from ter Steege et al. (2006) from more than 750 plots throughout Amazonia. The black line represents the observed abundance data and the blue line is the fitted log series with α = 71. The insert graph displays the same abundance data with genera binned into doubling classes of abundance, following Preston (1948). The red line is the best-fit lognormal distribution, fit to the data to the right of the mode. The lognormal fails to fit the distribution for rare genera, whose abundances are in the abundance classes to the left of the mode. The flat top of the distribution for rare taxa is characteristic of log series distributions for high-diversity communities.

Based on CTFS data and recent, very extensive plot data for Amazonia, there is reason to question the consensus view. One major challenge to the consensus view is the precise fit of Fisher's log series to the rank abundance curve of Amazonian tree genera (Fig. 3) (Hubbell et al. 2008). The log series says that the number of species having *n* individuals, ∅(*n*), in a sample is given by


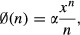
(1)

where α and *x* are parameters. Parameter α, known as Fisher's α, is a diversity parameter equal to the number of singleton species in the sample, and *x* is a number close to but slightly less than unity. Equation (1) bins species into abundance categories, but one can also fit the log series to rank abundance curves in which species are ordered in abundance from high to low abundance on the *x* axis, as in Figure 3.

In Figure 3, the observed abundance data are shown with the black line, and the smooth blue line is the best-fit log series. A good fit to the log series implies that there are a large number of rare and very rare species. The data come from over 750 plots located throughout Amazonia (data from ter Steege et al. 2006).

Neutral theory predicts that the log series is the steady-state distribution of relative species abundance on large biogeographic spatiotemporal scales when the size of populations at species origination is small (Hubbell 2001; Rosindell et al. 2010). Neutral theory derives the two parameters of the log series, *α* and *x*, from the per-birth speciation rate, the total size of the tree community in the biogeographic region, and the average per capita birth and death rates of species in the region (Volkov et al. 2003). Fisher's *α* and the biodiversity parameter *θ* of neutral theory are identical, so hereafter I refer to both as Fisher's *α*, which is about 71 for Amazonian tree genera. The other commonly used model of relative abundance, Preston's lognormal (Preston 1948), does not fit the data at all well. The insert graph is a Preston plot of the number of Amazonian genera binned into doubling classes (octaves) of abundance. Preston's lognormal (red line, insert graph) fits the upper half of the abundance distribution for common genera, but grossly underestimates the number of rare genera.

**Figure 4:**
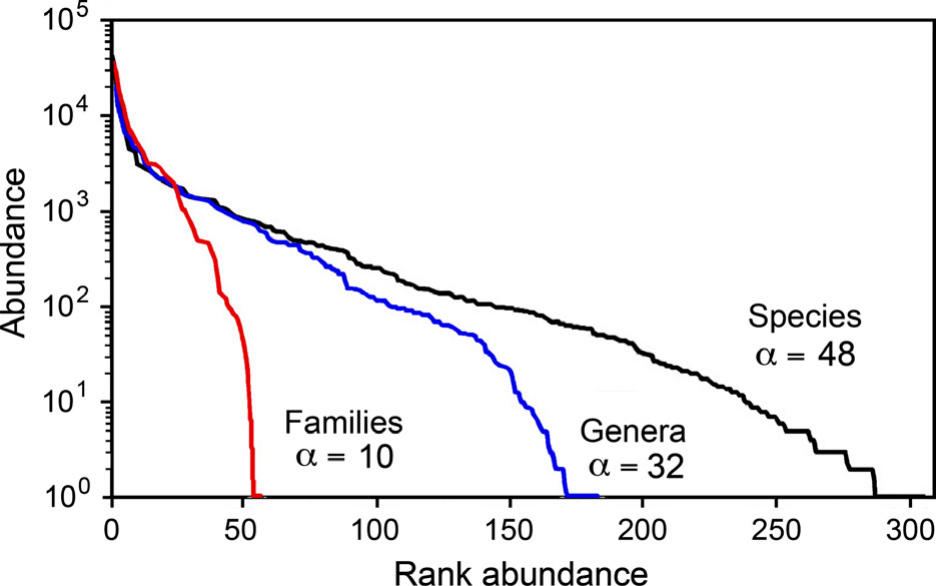
Rank-abundance curves Barro Colorado Island (BCI) trees and shrubs in the 50 ha BCI plot for three levels of taxonomic aggregation: species, genera, and families. As expected, Fisher's α becomes progressively smaller for higher taxonomic levels, reflecting the lower origination rates of the higher taxa.

In Hubbell et al. (2008), we fit the abundance distribution of Amazonian tree genera (Fig. 3), not species, but the use of genera does not pose any difficulty of interpretation of the data on rarity. We used genera because genera were more stable taxonomically than species-level taxa for Amazonian species at the time the paper was written (ter Steege et al. 2006). However, using genera is no problem because the theory fully accommodates aggregation of taxonomic units into higher levels of classification. For example, the same qualitative patterns of commonness and rarity appear in the rank abundance curves for species, genera, and families in the 50 ha plot on BCI (Fig. 4). As expected, the Fisher's *α* (or equivalently, parameter *θ* of neutral theory) becomes progressively smaller as the taxon level increases, which, according to theory, reflects the lower origination rates of the higher taxa relative to the lower taxa.

In 2008, we predicted that once species-level taxonomy was better resolved for Amazonian tree species, the log series would also fit the species-level data better than any other model of relative species abundance; and this prediction has now been confirmed (ter Steege et al. 2013). These new analyses increase the estimated species richness in the Amazon by over a quarter from our original estimate of 12,500 (Hubbell et al. 2008). ter Steege et al. (2013) enumerated 4970 tree species with stems >10 cm DBH in 1170 plots scattered all over Amazonia. Based on a very tight fit of the log series to their plot data, ter Steege et al. estimate that there are about 16,000 tree species in Amazonia.

Chao et al. (2009) developed a widely used statistic for sufficient sampling to estimate the asymptotic number of species minimally to be expected if collections were to continue, that is, including species not yet sampled. The method is based on an assumption that by increasing sample size, eventually all singleton species will become doubletons. However, this expectation does not hold for Fisher's log series: No matter how large a sample one takes, the log series predicts that the abundance category of singletons will remain the category with the most species. Using the data from ter Steege et al. (2013), one can easily show that the Chao estimator grossly underestimates the number of Amazonian tree species. The Chao estimator for the minimum number of unsampled species remaining to be collected is given by:


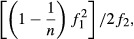
(2)

where 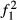 is the square of the number of singleton species, *f*_2_ is the number of doubleton species, and *n* is the total number of individuals sampled (*n* = 639,631). In the 1170 plots, there were 647 singleton species and 345 doubleton species (Supplemental Material, ter Steege et al. 2013). Because *n* is very large, the factor (1−1/*n*) is effectively unity in Expression (2), so the Chao estimator of the minimum unfound species reduces to the square of the number of singleton species divided by twice the number of doubleton species. This yields a Chao estimate of a minimum of about 607 species that remain uncollected. Because the total number of species observed in the 1170 plots was 4970, we obtain a total minimum estimate of 5577 species in Amazona using the Chao estimator. However, even if we accept that this is a valid minimum estimate of tree species richness in the Amazon, it falls far short of the actual number of Amazonian tree species recorded in herbaria, of which there are more than 12,500 (H. ter Steege, pers. comm.). Whatever the true number of Amazonian tree species is, there can be no longer any doubt that many of them are globally rare, not just locally rare. As one measure of absolute rarity to apply to ter Steege et al., one might arbitrarily define hyper-rare tropical tree species as those with fewer than 10^3^ individuals in total. By this yardstick, ter Steege et al. (2013) estimate that nearly 6000 tree species, about 37.5% of all Amazon tree species, are hyper-rare.

There are restrictive conditions under which the “all-species-are-everywhere” hypothesis might still be consistent with Fisher's log series. To be consistent requires that as the total abundance of a given species declines, the mean density of the species must also decline throughout its range. For example, consider a rare tree species with a total population size of 10^5^ individuals. If we expect to spread this species over the 7.2 × 10^6^ km^2^ of the Amazon Basin, this implies a mean density of one tree every 72 km^2^. For the 6000 hyper-rare species discussed above with fewer than 10^3^ individuals, the mean density would have to be less than one tree every 7200 km^2^. Most tropical trees reproduce sexually and are highly outcrossed (Bawa 1974), although tree density can affect the outcrossing rate (Murawski and Hamrick 1991). Thus, even rare species must occur at a minimum density for cross-pollination to occur. Also most tree species have limited seed dispersal distances. These facts imply that viable populations of rare species cannot be thinly spread over the entire Amazon Basin, and therefore must have more limited ranges than do common species. If rare species are also spatially aggregated within their ranges, such clumping would require that their total range size be smaller, and generally much smaller, than if they were distributed everywhere at their mean density within their range.

**Figure 5:**
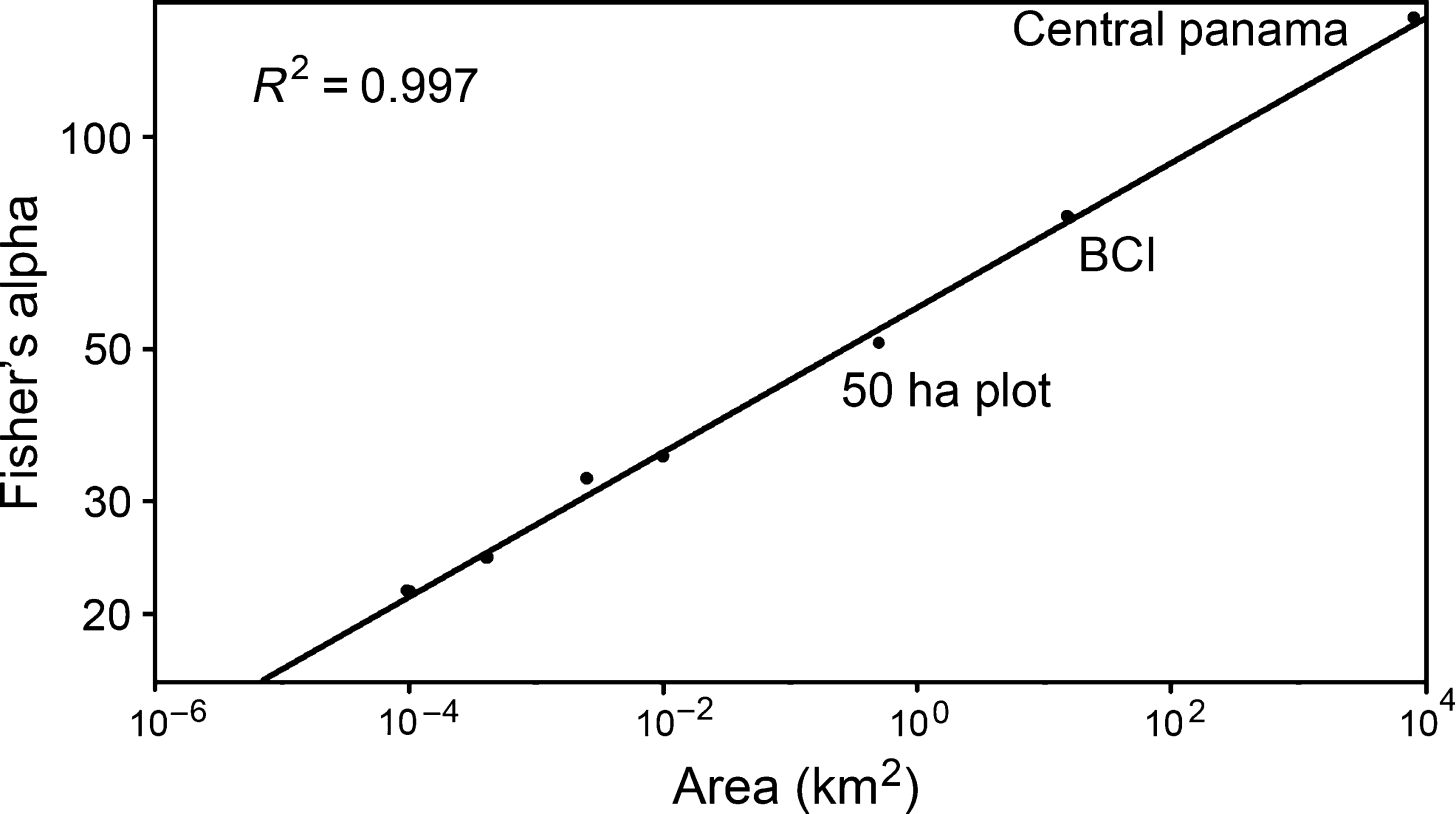
Power-law scaling of Fisher's α for tree communities in central Panama, based on data from the 50 ha plot on Barro Colorado Island (BCI), the well-known tree flora of Barro Colorado Island (Croat 1978), and on inventories of 40 tree plots distributed across the Isthmus of Panama (http://www.ctfs.si.edu) conducted by R. Condit and R. Perez. The equation of the power law is (Fisher's α) = 1.7099 + 0.1062 (Area, km^2^).

There is additional evidence against the “all-species-are-everywhere” hypothesis. Theoretically, Fisher's *α*, or equivalently, the biodiversity number *θ*, of the log series, should be constant and independent of sample size if the entire biogeographic region can be randomly sampled. In practice, Fisher's *α* increases slowly with increases in sample area. The fact that Fisher's *α* increases with sample area is inconsistent with – and indeed falsifies – the “all-species-are-everywhere” hypothesis. For example, consider the increase in Fisher's *α* in forests across central Panama: there is a power law relationship in the spatial scaling of Fisher's *α* (Fig. 5). The four small-area points in the figure are cumulative samples from within the 50 ha census plot on BCI. The point for all of BCI is estimated from the well-known tree flora of the island (Croat 1978) combined with estimates of the size of the total tree population on the island. The data point for Central Panama is based on a series of 40 h forest inventory plots, each 1 ha or larger, across the isthmus of Panama in which Condit et al. (2005) enumerated all trees ≥10 cm DBH. The power law is remarkably precise, with an exponent of 0.1062 and a coefficient of determination, *R*^2^ = 0.997. This power law currently has no theoretical explanation, but the precision of the relationship suggests that a theoretical connection exists between beta diversity and species abundance and distribution. When one extrapolates the power law in *α* observed for Central Panama to the size of a region equivalent to all of Amazonia, one underestimates the value of Fisher's *α* for species diversity expected for the Amazon Basin by about threefold (Hubbell et al. 2008). Potential factors contributing to an underestimate are that Panama is a narrow isthmus that is also relatively young geologically, factors which may have reduced the equilibrium diversity of central Panama's tree flora relative to the diversity that would be expected in a similar area sampled in a continuous continental landscape. This underestimate, however, only further underscores the conclusion from the power law Fisher's *α* – area relationship that trees species in lowland tropical forests are not distributed everywhere. Indeed, the evidence from the analysis of *β* diversity of neotropical tree communities (Condit et al. 2002) implies that there must be a positive correlation between the total abundance and the geographic range size of species. Such a correlation has been found by ter Steege et al. (2013) in many common Amazonian trees.

## The Distribution, Abundance, and Range Size of Tropical Tree Species

**Figure 6:**
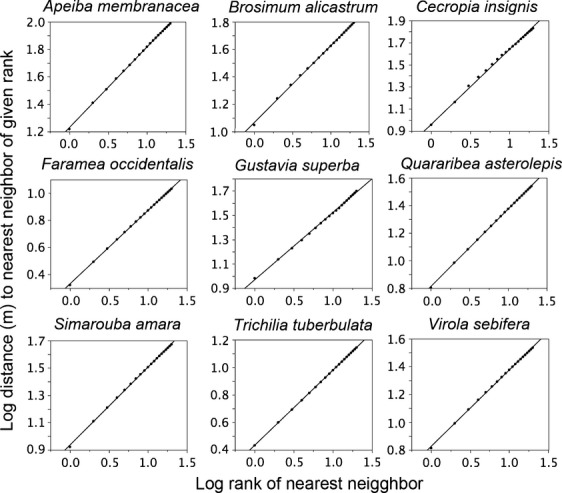
Nearest neighbor power laws for 9 Barro Colorado Island (BCI) species, relating the log of distance in m to the *n*th nearest neighbor to the log of the rank of nearest neighbor, illustrating the uniformly high coefficients of determination of these relationships for BCI species. All census size classes are included (stems >1 cm dbh). Species shown: *Apeiba membranacea* (Tiliaceae), canopy tree, moderately light demanding; *Brosimum alicastrum* (Moraceae), shade tolerant; *Cecropia insignis* (Cecropiaceae), canopy tree, very light demanding; *Faramea occidentalis* (Rubiaceae), understory tree, shade tolerant; *Gustavia superba* (Lecythidaceae), midstory tree, shade tolerant; *Quararibea asterolepis* (Bombacaceae), canopy tree, shade tolerant, *Simarouba amara* (Simaroubaceae), midstory tree, moderately light demanding; *Trichilia tuberculata* (Meliaceae), canopy tree, shade tolerant; *Virola sebifera* (Myristicaceae), midstory tree, shade tolerant. Of the 217 species analyzed having >20 individuals in the plot, the power laws had coefficients of determination >0.999 in 50.7% (110) species, and >0.99 in 88.5% (192) species.

**Figure 7:**
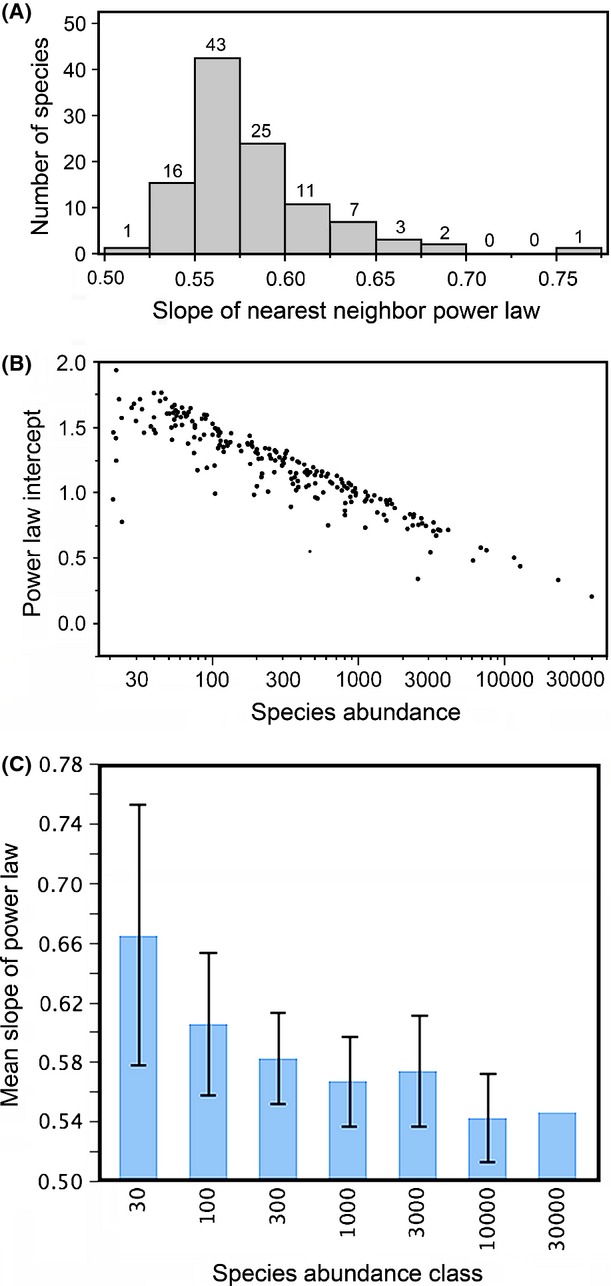
Distribution of the nearest neighbor power-law parameters. Panel A: Distribution of the slopes of the power laws. One expects a slope of 0.5 for randomly distributed species. Slopes >0.5 indicate aggregation. The modal slope category is between 0.55 and 0.60, indicating a moderate level of aggregation. Panel B: The relationship between log abundance of species and the intercept of the power law for the given species. The intercept is the first nearest neighbor distance. As expected, rare species exhibit greater first nearest neighbor distances than do common species. Panel C: The relationship between the slope of the nearest neighbor power law and the log abundance of species, binned into classes of log(10) abundance of width 0.5. Rare species have steeper slopes because nearest neighbors of order higher than the first are also more distant than in common species. The error bars are one standard deviation of the data, not of the mean.

Can we say anything about how tropical tree species are spatially distributed, and what the consequences of this spatial distribution are for species range sizes? It turns out that we can, and the CTFS plot data are very helpful in this regard. There is a power law relationship built into nearest-neighbor relationships for individual species (Hubbell et al. 2008). The logarithm of the distance to the *n*th nearest conspecific neighbor is linearly related to the logarithm of the rank of nearest neighbor, *n*. For a randomly distributed population, the expected distance to the *n*th nearest neighbor is proportional to the square root of the rank *n* of nearest neighbor, so the slope of the power law is 0.5. However, the power law relationship also holds for aggregated distributions. Hubbell et al. (2008) examined the relationships for nearest-neighbor distances in relation to rank nearest neighbor in BCI tree species, and found that all species exhibited power laws, regardless of taxon. A sample of species and their nearest-neighbor power laws is shown in Figure 6. The power laws are very precise, and about half of all species have coefficients of determination in excess of 0.999. These power laws hold for species regardless of differences in abundance, life history (both shade tolerant and shade intolerant species), and growth form (shrubs to canopy trees). The power laws in Figure 6 are for all stems >1.0 cm DBH, but power law relationships also hold for subsets of larger individuals, for example, for stems >20 cm DBH (Hubbell et al. 2008). Clumping of individuals in a population causes the slopes of the power law to be steeper than 0.5, the slope expected for a randomly distributed species. Figure 7A shows the distribution of slopes of the power laws of all species (217) having abundances ≥20 in the 50 BCI plot. Abundance affects especially the intercept (first nearest-neighbor distance) (Fig. 7B) but also the slope (Fig. 7C) of the power law relationship. Rare species generally have greater first nearest-neighbor distances, and the *n*th nearest neighbor is generally farther away in rare species than in common species.

Power laws take the complex spatial geometry of populations fully into account. There are tree species that have very patchy distributions in the BCI plot, and others that approach randomness, but in every case the power law holds. This is important because if power laws are very precise in the sense of having coefficients of determination very near 1.0, then they have the property of scale independence and one can extrapolate them to any arbitrary number of neighbors and spatial scale. Hence, if one knows the total global population size of a species, in principle one can calculate the distance to the last individual from the first individual, and thereby estimate range size.

The power law relationships are universally found in all BCI species within the 50 ha plot, but do they also hold on landscape to biogeographic scales? We have few examples of tropical tree species that have been stem mapped on larger spatial scales, but James Kellner of Brown University has used Quickbird satellite images of BCI to map three canopy tree species over the entire 15 km^2^ of BCI: *Dipteryx panamensis* (Fabaceae), *Jacaranda copaia* (Bignoniaceae), and *Handroanthus* (formerly *Tabebuia*) *guayacan* (Bignoniaceae). The mapping was possible because each of these canopy emergent species flowers synchronously for a few weeks, the flowering crowns have distinctive spectral signatures, and satellite images are available for multiple years during the peak flowering intervals of each species. For these three species, mapped over the entirety of BCI, the nearest-neighbor relationships are all very precise power laws. Table 1 presents a comparison of the parameters of the power laws fit to the subset of the populations within the 50 ha BCI mapped plot, and for the entire island, and for all stems >1 cm DBH, and all stems >20 cm DBH. As might be expected, the intercept values all increase for the regressions for trees >20 cm DBH because in general saplings >1 cm DBH are closer together than adult trees. We cannot precisely compare the power laws for trees >20 cm DBH in the 50 ha plot with the trees mapped by remote sensing, but most of the remotely sensed trees are large. Despite this, the power law intercept and slope values for trees >20 cm DBH and for remotely sensed “canopy” trees are quite similar. Two conclusions result: First, for the available data on landscape scales of mapped tree populations, the power law relationship holds for all species. Second, there is consistency between measurements taken in a 3% sample (the 50 ha plot) of a reasonably large landscape (the 15 km^2^ area of BCI), indicating that power law mesoscale extrapolation is valid for at least these three tropical tree species.

**Table 1:** Data on power law relationships for three species, *Dipteryx panamensis* (Fabaceae), *Jacaranda copaia* (Bignoniaceae), and *Handroanthus* (formerly *Tabebuia*) *guayacan* (Bignoniaceae). Parameters of the power laws estimated from population samples of these three species within the 50 ha plot are similar to the parameters of the power laws estimated from the populations of these species mapped over the 15 km^2^ entirety of Barro Colorado Island (BCI). The populations for the whole islands were visible in the canopy by remote sensing, but the exact stem diameters of these trees are not known. Data courtesy of James Kellner, Brown University

Species	Scale	Size (dbh)	Abundance	Intercept	Slope	*R*^2^
*Dipteryx panamensis*	50 ha plot	>1 cm	56	1.6296	0.6478	0.9970
	50 ha plot	>20 cm	28	1.8327	0.6673	0.9878
	15 km^2^ (BCI)	“Canopy”	744	1.7099	0.6586	0.9980
*Jacaranda copaia*	50 ha plot	> 1 cm	343	1.1549	0.6435	0/9995
	50 ha plot	> 20 cm	158	1.4000	0.6133	0.9992
	15 km^2^ (BCI)	“Canopy”	832	1.8649	0.6273	0.9981
*Handroanthus guayacan*	50 ha plot	>1 cm	76	1.4297	0.7679	0.9884
	50 ha plot	>20 cm	24	1.9046	0.6572	0.9843
	15 km^2^ (BCI)	“Canopy”	791	1.7099	0.6586	0.99868

On the assumption that nearest-neighbor power laws apply at even larger scales, we now jump to the problem of estimating species range sizes for rare tree species, assuming that we can extrapolate the power law patterns found on BCI. We can estimate range size for a species if we can determine its total global population size. If, as I argue in the preceding section, total tree species abundances are distributed as a log series in a large biogeographic region, then one can estimate the range sizes of species in the region.

Why are range sizes important? Range sizes of tropical tree species, particularly rare species, matter considerably to conservation strategies. If a particular tropical tree species is local and restricted to a small geographic area, this species is at greater risk to extinction both from habitat loss and climate change than a species having a broader geographic and climatic extent.

## Estimating Extinction Risk to Habitat Loss in Amazonian Tree Species

Several years ago, in the study referenced above (Hubbell et al. 2008), we attempted to estimate the numbers of tree species at risk to extinction in the Brazilian Amazon. Of course this is a rather speculative enterprise at present because the geography of tree species distributions in the Amazon remains poorly known – as indeed even the alpha taxonomy is. Nevertheless, our exercise was well worth attempting, in part to stimulate interest in the implications of tree species rarity and restricted range sizes for conservation and to encourage the collection of better data to answer the question more precisely. We estimated the range sizes of Amazonian tree species based on their abundances calculated from Fisher's log series. The very precise fit to the log series for abundances of tree genera (Fig. 3), and more recently for tree species (ter Steege et al. 2013) justifies the assumption that a log-series distribution must also describe the abundances of Amazonian tree species. With the log-series species abundances in hand, we can use the power law relationships for nearest neighbors to estimate the distribution of range sizes. We used the average slopes and intercepts of the power laws obtained from BCI tree species, adjusted for tree abundance (Fig. 7). We then superimposed the species ranges so calculated on land-use maps of the Brazilian Amazon prepared by Laurance et al. (2001), who classified land into four categories: “pristine”, and light-, moderate-, and heavy-impact areas. The heavy-impact areas were essentially completely deforested and converted into agricultural or urban use. Laurance et al. presented two scenarios, one “optimistic”, and one “pessimistic”, showing projections of the amount of land in each category by mid-21st century. We based our estimates of extinction on the assumption that if the range of a species fell entirely within heavy-impact areas, it was at high risk of extinction.

We computed species range sizes as follows. Based on the richness of the tree flora, which contained, by our 2008 calculation, an estimated 11,210 tree species in the Brazilian portion of the Amazon, and the estimated total number of trees >10 cm DBH in the Brazilian Amazon, 2.68 × 10^11^, we obtain a value of Fisher's *α* of 500 (for the full Amazon Basin, *α* is larger: 743). We based our estimate of the number of trees on the average tree densities in the >750 plots across Amazonian (600 per ha) that were available at the time of our analysis. This density yields a reasonable estimate, averaging the lower density of trees in eastern, drier Amazonian forests of about 400 trees per ha >10 cm DBJ, and the higher densities of about 700 per ha in western, wetter Amazonian forests (the CTFS plot in Yasuni National Park in Ecuador has 701 trees per ha >10 cm DBH).

The resulting function for range size is a power law of abundance, and might be expected from the preceding discussion. The predicted functional relationship between range size in square kilometers and species abundance is as follows:


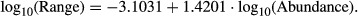
(3)

**Figure 8:**
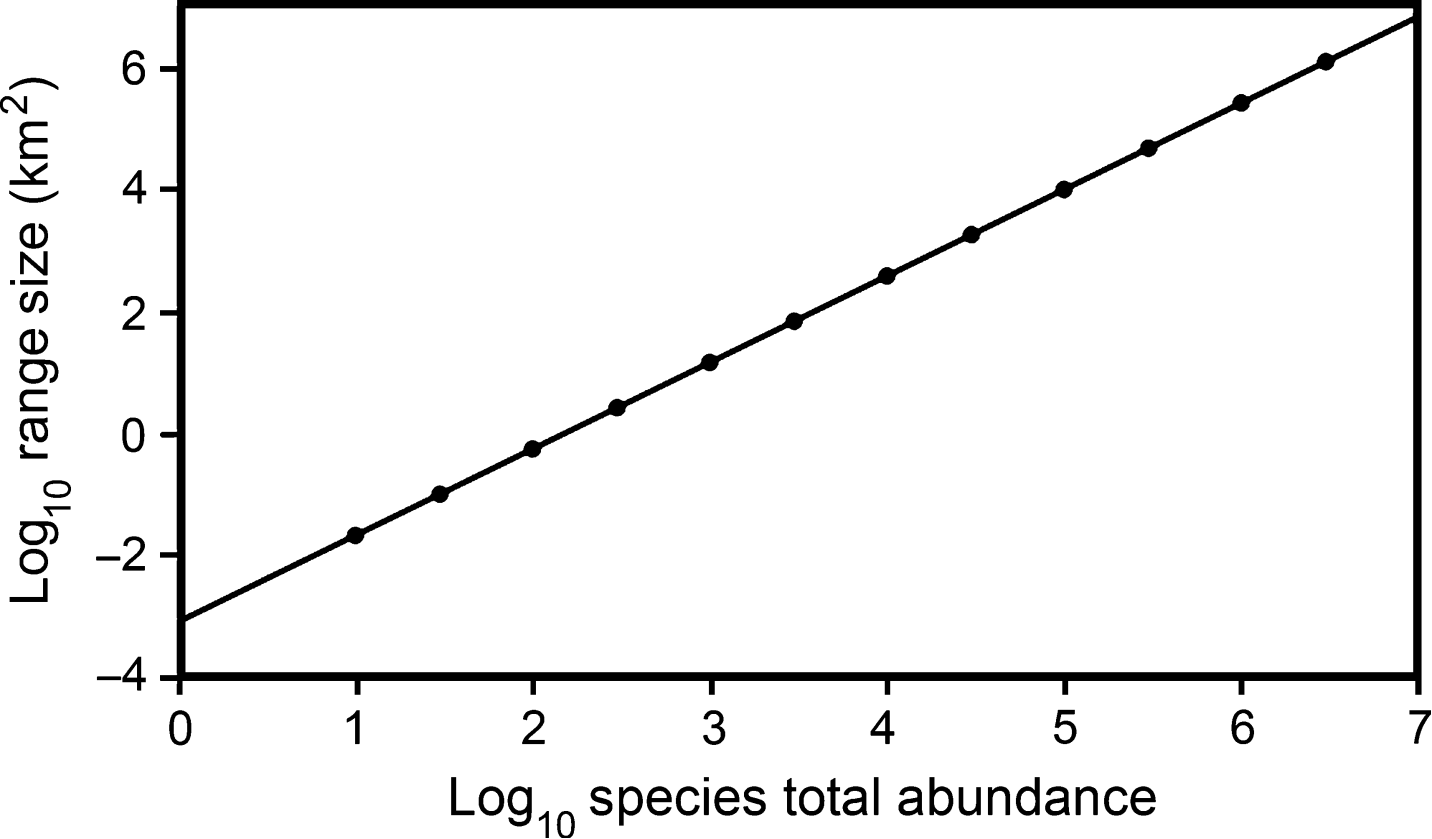
Power-law describing the functional relationship between range size in km^2^ and total abundance of species, derived from the power-law relationships of tree species on Barro Colorado Island. The slope of the relationship is quite steep (1.4), indicating that range size increases much faster than linearly with increasing abundance.

I have graphed Equation (3) in Figure 8. It is instructive that the size of species ranges is not a linear function of species abundance, as might have been expected from a simple crude density argument. The slope of the power law is quite steep (1.42), and means, for example, that a 10-fold increase in abundance results in a 26.3-fold increase in range size. The intercept value for an abundance of a single individual is also of the right order of magnitude, 7.89 × 10^−4^ kn^2^, which corresponds to an area 28 m on a side.

From these calculations and based on the estimated species-level log-series distribution of species abundance in the Brazilian Amazon, we estimated that the most abundant tree species in this portion of the Amazon Basin has a total abundance of 3.89 billion individuals; but despite its high total abundance, this species represents only 1.4% of all trees. With improved data on the species-level log series, ter Steege et al. (2013) arrived at a very similar estimate for the abundance of the most common species in Amazonia. Many tree species are geographically widespread and abundant: An estimated 3248 species (29.0%) in our 2008 analysis have more than a million individuals >10 cm DBH. At the rare end of the abundance distribution, however, we estimated that more than a third of all species (3981 or 35.5%) have total population sizes of <10^3^ individuals, and are thus extremely rare. If the current estimate of total tree species richness in Amazonia had been available to us in 2008, we would have estimated 5680 hyper-rare species, very close to the revised estimate of 6000 hyper-rare species obtained by ter Steege et al. (2013), working with an expanded and updated data set having 57% more plots. If our range size estimates are reasonably accurate, then these hyper-rare species are expected to occupy ranges of <15 km^2^, a minute fraction of the total area of the Amazon Basin, making them extremely difficult to find.

Based on the map of the four land-use categories provided by Laurance et al. (2001), we estimated that about half of the species in the Brazilian Amazon with populations below a total abundance of 10^4^ individuals could be at high risk to extinction by midcentury, or have actually already gone extinct from habitat loss, under the Laurance pessimistic scenario (Hubbell et al. 2008). Under the optimistic scenario, the extinction estimate dropped, but not greatly, to about 35% of species with abundances <10^4^. However, we predicted no extinctions from habitat loss for species with abundances in excess of 10^6^ individuals because of their large range sizes. The total predicted percentage loss of all 11,210 species under the pessimistic scenario was 23.3%, and under the optimistic scenario was 16.3%.

## Discussion

There are many uncertainties regarding these extinction estimates, and they need to be considered as crude approximations because of these uncertainties. One caveat is that we do not know for certain whether the log series truly characterizes relative species abundances in the Amazon, despite the extremely precise fit to existing data from a large set of small plots throughout the Amazon Basin (ter Steege et al. 2013), and the CTFS plot data. It is likely that many species locally rare in the CTFS and Amazonian plots are common in places not yet sampled. In surveys of herbaria, R. Condit (pers. comm.) has found that almost all CTFS tree species are known from multiple geographic sites. There is evidence of patchy commonness in the distributions of the three BCI species that Kellner mapped by remote sensing over all of BCI (Table 2). Mean densities are <1 canopy tree/ha for all three species, and the species are absent from two thirds to three quarters of all hectares on the island. However, in a very small fraction of hectares, abundances are larger. For example, the abundances of the species are ≥5 trees/ha in 12 (0.89%), 2 (0.13%), and 13 (0.87%) hectares of 1500 total BCI hectares for *Dipteryx*, *Jacaranda*, and *Handroanthus,* respectively. Moreover, the hectares with more than one tree are themselves aggregated (J. Kellner, unpubl. data). This patchiness, however, does not invalidate our earlier power law analyses of range size, which as I pointed out above, fully take into account the nonrandom spatial distributions of species.

**Table 2:** Landscape abundance patterns of *Dipteryx panamensis* (Fabaceae), *Jacaranda copaia* (Bignoniaceae), and *Handroanthus* (formerly *Tabebuia*) *guayacan* (Bignoniaceae) across 1500 ha on Barro Colorado Island, Panama (data courtesy of James Kellner). The maximum number of trees/ha of any of these species was in *Tabebuia* (12)

No. Canopy trees/ha	*Dipteryx panamensis*	*Jacaranda copaia*	*Handroanthus guayacan*
0	963 (64.20%)	1190 (79.33%)	983 (65.53%)
1	315 (21.00%)	239 (15.93%)	348 (23,29%)
2	137 (9.13%)	46 (3.97%)	113 (7.53%)
3	51 (3.49%)	21 (1.44%)	25 (1.67%)
4	22 (1.47%)	2 (0.13%)	18 (1.20%)
5	7 (0.47%)	1 (0.06%)	4 (0.27%)
6	1 (0.06%)	1 (0.06%)	5 (0.33%)
7	2 (0.13%)	0	2 (0.13%)
8	1 (0.06%)	0	0
9	1 (0.06%)	0	1 (0.06%)
10	0	0	0
11	0	0	0
12	0	0	1 (0.06%)
Mean, no./ha	0.602	0.275	0.537
Variance, no./ha	1.057	0.394	0.989

The most important question remains: Are there lots of extremely rare species, as predicted by the precise fit of Fisher's log series? We do not know the answer to this question. There has been much debate in the literature about whether to expect so many rare species, particularly as they would tend to have extremely short evolutionary life spans (Ricklefs 2003). But this issue can be resolved if one postulates that speciation is a protracted process, and that completion of speciation only takes place after an incipient species has achieved some appreciable global abundance (Rosindell et al. 2010). In practice, all species exhibit intraspecific variation, and recognizing lineages destined to become new species before speciation occurs is nontrivial if not impossible in general. The ambiguities of what to recognize as species is not without conservation implications, just as is the problem of conserving intraspecific variation and subspecies.

A second caveat about the findings of Hubbell et al. (2008) are the assumptions we made about species responses to the land-use categories of Laurance et al. (2001), which remain essentially unknown. We made what we thought was a reasonable assumption that portions of the Brazilian Amazon that were “heavily impacted”, essentially completely deforested, would lose species whose ranges were restricted to heavy-impact areas. However, some small forest fragments still remain in these areas, and we do not know how many species have persisted or will survive in the future in them.

Finally, one of the limitations of Hubbell et al. (2008) was that we did not have data on where actual species were distributed, so in the simulations, we distributed species’ ranges at random across the Laurance land-use maps. Based on presence–absence data from the databases of the Global Biodiversity Information Facility (GBIF), Feeley and Silman (2009) revised our estimates of species at high extinction risk. One of their main points was that there is a steep biodiversity gradient from west to east across the Brazilian Amazon, with many more species on the piedmont of the Andes. Second, they noted that much of the area classified as heavy-impact areas in the eastern regions of the Brazilian Amazon either had limited forest cover or had been deforested for several centuries. Thus, one would overestimate future extinctions by using our random placement model. Third, they used GBIF data to estimate species ranges, and did not find the large number of rare species with restricted ranges that are predicted by the log series. Incorporating these factors, Feeley and Silman (2009) came up with a range of estimates of 5–9% of species “committed to extinction” by 2050, significantly fewer than the estimates of 16–23% that we obtained.

Given the west–east gradient in species diversity, the random placement model we used overestimates extinctions, and so Feeley and Silman's study is a welcome improvement. However, I am less convinced by the range size estimates derived from GBIF data. There is almost certain to be an unavoidable sampling bias against finding and collecting rare and local species. This is in spite of the fact that ter Steege et al. (2011) has demonstrated that plant collectors favor rare species, and tend not to collect common species in proportion to their abundance. This collector bias does not address the lack of collections over much of the Amazon. Collections are restricted in most cases to relatively easily accessible areas near roads and rivers. More remote areas are much more poorly sampled. Feeley and Silman (2011) analyzed over 800,000 collection records, and found that >80% of species had <20 collection records. It would be a valuable exercise to rarify the GBIF data and ask the question: How sensitive are estimates of range size to sample size (number of collections)? Also, there are many errors of identification in the GBIF data; and the taxonomic instability of species remains a major problem with lumping and splitting. If lumping tends to outweigh splitting, the numbers of rare species will be underestimated; whereas if splitting outweighs lumping, there will be too many rare species. However, the new evidence on species-level abundances of Amazonian trees (ter Steege et al. 2013) strongly supports the Fisher log series model of species abundance; these, analyses are plot-based, rather than the collector GBIF analyses by Feeley and Silman (2011), data that are collector-based, not plot-based.

## Conclusion: Conservation Challenges Posed by Rare Tropical Tree Species

Whatever the true distribution and abundance of rare tropical tree species, such species pose special problems for conservation. If rare species typically have relatively small ranges, then ensuring that they are protected in reserves will be a challenge, especially if rare species are not spatially codistributed over the landscape. Recent improvements in remote-sensing technology, particularly using airborne LiDAR combined with hyperspectral data with spatial resolution under 1 m^2^, may offer enhanced opportunities to map tree species over wide regions of the tropics, and resolve many of the current uncertainties about the distribution of rare tropical tree species. Many more tropical tree species than previously thought have unique spectral signatures in canopy reflectance (G. Asner, pers. comm.). However, obtaining adequate reflectance samples of rare species remains a challenge, and these remotely made reflectance readings will need to be calibrated against ground measurements.

Perhaps an even bigger challenge to conserving tropical rain forests are the novel climates forecast later this century for much of the tropics (Williams et al. 2007). It is not at all clear that the changes in annual temperature and precipitation predicted by 2080 in the Amazon Basin, as well as changes in seasonality, lie within the evolved climate envelopes of most tropical tree species (Feeley and Silman 2010). It may well be that geographically widespread species will exhibit greater phenotypic plasticity and fare better if their populations have been exposed to a greater range of climatic conditions than species having geographically more restricted ranges. Given that a relatively small number of tree species (in the low hundreds) comprise most of the tree individuals in Amazonian forests (ter Steege et al. 2013), climate change may exacerbate the extinction of rare species due to deforestation, reducing tropical forests largely to a residual diversity of these widespread, hyperabundant tree species.

To the recent discussion of the urgent need to fill the “data void” on responses of species to climate change (Feeley and Silman 2011), I would add that we also urgently need better data on the distribution of rare tree species in tropical forests. Obtaining these data is no mere academic exercise, but has profound implications for conservation of tree diversity in tropical forests, and should be an immediate international research priority for taxonomists, ecologists, and biogeographers.
